# A Case Report of Moyamoya Disease Presenting as Headache in a 35-year-old Hispanic Man

**DOI:** 10.7759/cureus.4426

**Published:** 2019-04-10

**Authors:** Daniel B Azzam, Ajay N Sharma, Ekaterina Tiourin, Alvin Y Chan

**Affiliations:** 1 Neurosurgery, University of California, Irvine, USA; 2 Dermatology, University of California, Irvine, USA; 3 Miscellaneous, University of California, Irvine, USA

**Keywords:** moyamoya, headache, hemorrhage, stroke, neurosurgery

## Abstract

Moyamoya disease (MMD) is a rare, chronic vaso-occlusive disease affecting the arteries of the Circle of Willis, leading to the development of characteristic collateral vessels. In this paper, we present a case of a 35-year-old Hispanic male who presented to the emergency department with new onset headaches. On examination, Glasgow Coma Scale score was 3T. The patient was investigated with head CT scan and cerebral angiogram, diagnosed as MMD, and treated with emergent ventriculostomy. Ultimately, the patient underwent extracranial-intracranial (EC-IC) bypass surgery for treatment of Moyamoya.

## Introduction

Moyamoya disease (MMD) is a progressive cerebrovascular disorder characterized by progressive stenosis of the terminal portions of the intracranial internal carotid arteries due to hypertrophy of smooth muscle in the vessel walls [1-4]. Reduced blood flow to the brain leads to the growth of collateral vasculature such as branches from small leptomeningeal vessels. Imaging of the collateral vasculature was characterized in Japan as a “puff of smoke,” or moyamoya in Japanese. Incidence of MMD is the highest in Japan, where there are three cases per 100,000, and in females (2:1 ratio) [2]. MMD is now seen throughout the world with a bimodal age of onset, with children presenting around age 5 and adults presenting around age 40 [2]. The presentation in a child is typically a stroke or a transient ischemic attack, while in adults it is typically a hemorrhage. Less commonly, MMD may present with a headache or a seizure. MMD affects bilateral internal carotid arteries, whereas a unilateral presentation of the same underlying pathology is known as Moyamoya syndrome and is associated with conditions like Down syndrome, neurofibromatosis type 1, and sickle cell disease.

## Case presentation

A 35-year-old Hispanic male presented to the emergency department for sudden onset worsening headaches over the past four days. Headaches were diffusely felt, not localized to a specific head region, and not relieved by over the counter pain medication. There was no associated trauma, fever, night sweats, loss of consciousness, photophobia, neck stiffness, or visual disturbances. His past medical history was significant for migraine headaches, type 2 diabetes mellitus, and benign essential hypertension. Physical exam revealed Glasgow Coma Scale score of 3T on 15L of oxygen, pupils 2-2.5 mm bilaterally, inability to arouse by voice or painful stimulation, and paralysis of upper and lower extremities bilaterally. Vitals include (heart rate = 89/min, blood pressure = 159/78 mmHg, temperature = 37°C, respiratory rate = 22/min, and oxygen saturation = 99% on 15L of oxygen). Labs were significant for respiratory alkalosis (blood pH 7.54, pCO2 27.5 mmHg), normal oxygenation (159.3 mmHg), and negative urinary toxicology screen.

**Figure 1:**
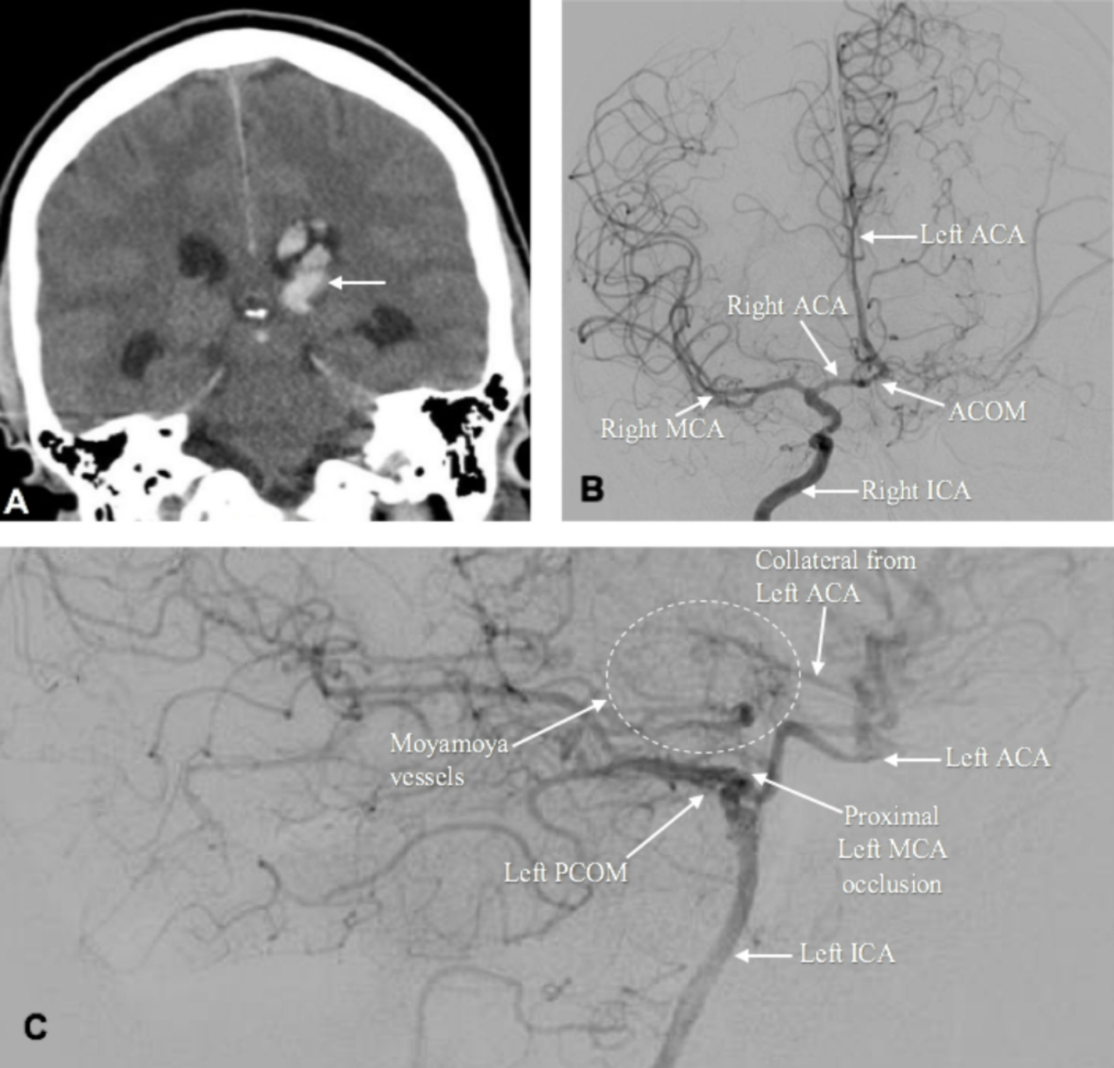
Panel A: noncontrast head CT showing rupture of the left thalamic hematoma into the lateral ventricle; Panel B: cerebral angiogram A-P view demonstrating right ICA injection showing the right ICA supplying the left ACA via the ACOM, as well as moderate right ICA stenosis; Panel C: cerebral angiogram lateral view demonstrating left ICA injection showing occlusion of the left ICA, as well as the left MCA territory being collateralized through leptomeningeal collaterals from the left ACA, which is supplied by the right ICA via the ACOM. Moyamoya vessels are present in the left basal ganglia area, left thalamic area, and left ethmoidal area. ICA, internal carotid artery; ACA, anterior cerebral artery; MCA, middle cerebral artery; ACOM, anterior communicating artery; PCOM, posterior communicating artery.

A head CT scan revealed a 1.5 cm hematoma in the left thalamus with expansion into the lateral ventricle (Figure 1A). Cerebral angiogram demonstrated complete stenosis of the left internal carotid artery (ICA) and partial stenosis of the right ICA (Figures 1B-1C). Diagnostic studies coupled with clinical presentation were compatible with a diagnosis of MMD. Patient treatment was initialized with emergent bedside right ventriculostomy (with opening pressure of 11 cmH2O) for intracranial pressure monitoring and for diversion of cerebrospinal fluid and hematoma. This patient ultimately underwent surgical revascularization. The procedure included a left superficial temporal artery-left middle cerebral artery bypass without any complications.

## Discussion

Diagnosing MMD may be challenging but should be guided by its typical presentation, underlying pathology, and appropriate angiography studies. MMD is a cerebrovascular disorder characterized by progressive stenosis of the internal carotid arteries at their terminal portions [1-2]. As the arteries gradually occlude, a compensatory collateral network of leptomeningeal vessels in a “puff of smoke” formation develops to bypass the blockage. Patients who have these characteristic Moyamoya vascular findings, along with an underlying associated condition or a strongly associated risk factor such as radiotherapy to the head and neck (often for optic gliomas, or pituitary tumors) are classified as having Moyamoya syndrome. Patients without these known associated risk factors are said to have MMD, which is more common and is a bilateral disease. Therefore, a unilateral presentation is considered Moyamoya syndrome, and 40% of these patients will develop contralateral disease as well.

The incidence of MMD is the highest in Japan, but has now been reported in ethnicities across the world, such as the Hispanic male we present here. Interestingly, the disease is twice as common in females compared to males. MMD has a bimodal age of presentation. Children around 5 years of age present with symptoms of brain ischemia, often triggered by hyperventilation (e.g. transient ischemic attacks, ischemic strokes, neurological deficits, and alterations in consciousness). Adults present in their mid-40s with recurrent headache and intracranial hemorrhage, often in the subcortical brain structures such as the basal ganglia. The hemorrhage is likely due to rupture of fragile collateral vessels or the development of cerebral aneurysms from shear stress; headaches may be due to dilated leptomeningeal collateral vessels stimulating nociceptors in the dura.

Surgical revascularization is the primary treatment of MMD. Previous studies have estimated that without treatment, around two-thirds of Moyamoya patients have progression of symptoms over five years, with poor outcome [2]. However, after surgical treatment, symptomatic progression is reduced to only 2.6%, with almost all patients remaining stroke free at a time point of five years after surgery [5-8]. The present patient discussed above was treated successfully with a direct extracranial-intracranial (EC-IC) arterial bypass surgery. The surgical revascularization options can be split into direct, indirect, and combined approaches. The direct EC-IC bypass performed here is commonly used in adult patients. The indirect encephalo-duro-arterio-syangiosis (EDAS) procedure involves laying down a branch of the superficial temporal artery onto the brain surface, allowing for neovascularization to supply blood flow to the tissue indirectly rather than a direct anastomosis. The EDAS procedure is more commonly performed in children.

## Conclusions

Our case brings forth the importance of considering MMD in the differential diagnosis for patients suffering from recurrent headaches in their third or fourth decade, as well as in children around age 5 who have refractory headaches or signs of ischemia. This case also highlights a rare presentation in a Hispanic male individual, despite the disease predominantly affecting females of Asian heritage. In general, when working up a patient for headache with neurological deficits, the differential diagnosis should include transient ischemic attack or ischemic stroke (e.g. atherosclerosis, thromboembolism, inflammation or vasculitis, sickle cell disease, MMD), intracranial hemorrhage (aneurysm, trauma, MMD), or mass lesion (e.g. tumor, arteriovenous malformation).

## References

[1] Miyamoto S, Yoshimoto T, Hashimoto N (2014). Effects of extracranial-intracranial bypass for patients with hemorrhagic moyamoya disease: results of the Japan Adult Moyamoya Trial. Stroke.

[2] Scott RM, Smith ER (2009). Moyamoya disease and Moyamoya syndrome. N Engl J Med.

[3] Kuroda S, Houkin K (2008). Moyamoya disease: current concepts and future perspectives. Lancet Neurol.

[4] Huang S, Guo ZN, Shi M, Yang Y, Rao M (2017). Etiology and pathogenesis of Moyamoya disease: an update on disease prevalence. Int J Stroke.

[5] Fung LW, Thompson D, Ganesan V (2005). Revascularisation surgery for paediatric moyamoya: a review of the literature. Childs Nerv Syst.

[6] Gudepu RK, Mohtashim AQ, Ihtesham AQ, Lakshman R (2014). Case report: a case report of Moyamoya disease in a 36 year old African American woman. F1000Research.

[7] Scott RM, Smith JL, Robertson RL, Madsen JR, Soriano SG, Rockoff MA (2004). Long-term outcome in children with moyamoya syndrome after cranial revascularization by pial synangiosis. J Neurosurg Pediatr.

[8] Choi JU, Seok Kim D, Kim EY, Lee KC (2002). Natural history of Moyamoya disease: comparison of activity of daily living in surgery and non surgery groups. Clin Neurol Neurosurg.

